# Clinical Implications of *Helicobacter pylori* Antibiotic Resistance in Italy: A Review of the Literature

**DOI:** 10.3390/antibiotics11101452

**Published:** 2022-10-21

**Authors:** Enrico Celestino Nista, Antonio Pellegrino, Lucia Giuli, Marcello Candelli, Tommaso Schepis, Sara Sofia De Lucia, Veronica Ojetti, Francesco Franceschi, Antonio Gasbarrini

**Affiliations:** 1 Department of Medical and Surgical Sciences, Università Cattolica Sacro Cuore, Fondazione Policlinico Universitario A, Gemelli IRCCS, 00168 Rome, Italy; 2 Department of Emergency, Anesthesiological, and Reanimation Sciences, Università Cattolica Sacro Cuore, Fondazione Policlinico Universitario A, Gemelli IRCCS, 00168 Rome, Italy

**Keywords:** *Helicobacter pylori*, antibiotic resistance, eradication therapy, gastritis

## Abstract

*Helicobacter pylori* (*H. pylori*) resistance to antibiotics has increased worldwide in recent decades, especially to clarithromycin. As a result, the World Health Organization (WHO) identified clarithromycin-resistant *H. pylori* as a “high priority” pathogen in 2017. As international guidelines recommend empirical therapy as first-line treatment, it is crucial to know local resistance rates and history of antibiotic use to determine the most appropriate first-line antibiotic treatment. Italy is one of the European countries with the highest prevalence of *H. pylori* infection and the highest percentage of antibiotic-resistant *H. pylori*. The aim of this review is to summarize all data on *H. pylori* antibiotic resistance in Italy in order to quantify the current rate and determine the most effective therapeutic approach. The study confirms an elevated level of resistance to clarithromycin, metronidazole, and levofloxacin in Italy. In addition, our results show a satisfactory eradication rate for a bismuth-based regimen when used as first- or second-line treatment. Naive patients are also successfully treated with clarithromycin-based quadruple therapies. Considering the good results of bismuth-based therapy as recovery therapy, this argues for the potential use of clarithromycin quadruple therapy as a first-line treatment.

## 1. Introduction

*Helicobacter pylori* (*H. pylori*) is one of the most widespread human pathogens, affecting approximately 4.4 billion people worldwide. Its prevalence is closely related to socioeconomic and hygienic conditions, as shown by the higher prevalence in developing countries (80%) compared to developed countries (20%) [1,2,3]. *H. pylori* is the leading cause of chronic active gastritis and, if left untreated, can lead to complications such as gastric ulcers, duodenal ulcers, atrophic gastritis, mucosa-associated lymphoid tissue (MALT) lymphoma, and gastric adenocarcinoma. In addition, other conditions such as unexplained iron deficiency anemia, idiopathic thrombocytopenic purpura, and vitamin B12 deficiency have been associated with *H. pylori* infection [4,5,6]. There are several genes that play important roles as virulence factors in different pathogenic strains of *H. pylori*, with the cagA and vacA genes being the best studied. Infection can be diagnosed by noninvasive (without endoscopy) and invasive (with endoscopy) tests. Each test has its limitations, which is why there is no single test as the gold standard. The noninvasive tests are the ^13^C-urea breath test (UBT), stool antigen test (SAT), and serologic test. Among the noninvasive tests, the UBT and SAT are first-line diagnostic approaches with higher accuracy than serological tests. The invasive tests are based on culture, rapid urease test (RUT), histology, and molecular biology, e.g., polymerase chain reaction (PCR), performed on biopsy specimens. PCR is the test that has shown the highest accuracy [7]. In 1994, the International Agency for Research on Cancer (IARC) classified *H. pylori* as a group I carcinogen for gastric adenocarcinoma [8]. *H. pylori* is the major risk factor for gastric cancer, with approximately 89% of non-cardia gastric cancers associated with chronic *H. pylori* infection [9]. Therefore, eradication of *H. pylori* is critical to reducing both the incidence and mortality of gastric cancer [10,11]. The Kyoto consensus states that all individuals infected with *H. pylori* should be treated unless other conditions such as comorbidities, local reinfection rates, competing community health priorities, and financial costs are present [12]. Treatment of *H. pylori* remains a challenge, as antibiotic resistance is an increasing problem for eradication [13]. For several years, the standard treatment was triple therapy with a proton pump inhibitor (PPI), amoxicillin, and clarithromycin or metronidazole. However, over the past decade, increasing rates of resistance to clarithromycin have led to a dramatic decline in efficacy, which is now below 80% in many countries [14]. For this reason, in 2017, the World Health Organization (WHO) classified clarithromycin-resistant *H. pylori* P as a “high priority” pathogen for the development of new antibiotics [15].

In addition, increasing resistance to fluoroquinolones and metronidazole also plays a critical role in the effectiveness of eradication measures [16]. To date, rates of primary and secondary resistance to clarithromycin, metronidazole, and levofloxacin exceed 15% worldwide [1].

In areas with high rates of clarithromycin resistance (>15%), current international guidelines recommend bismuth quadruple therapy (PPI, bismuth, tetracycline, and metronidazole) or concomitant non-bismuth quadruple therapy (PPI, clarithromycin, amoxicillin, and metronidazole) for 14 days as initial treatment [17,18,19].

In regions with high rates of resistance to both clarithromycin and metronidazole (>15%), bismuth quadruple therapy is the treatment of choice. Triple therapy with clarithromycin is recommended only when local resistance to this antibiotic is less than 15% and in patients who have not previously taken macrolides. In contrast, triple therapy with PPI, amoxicillin, and levofloxacin (PAL) is not recommended as first-line therapy [17,18,19].

Therefore, local resistance rates and history of antibiotic use are critical in determining the appropriate empiric antibiotic therapy [20]. After failure of first-line therapy, current guidelines recommend bismuth quadruple therapy or levofloxacin triple therapy (PPI, amoxicillin, levofloxacin) as second-line therapies [21,22]. Treatment should be based on culture or molecular testing after failure of second-line therapy [23]. The prevalence of primary antibiotic resistance in Europe was recently reported to be 18% for clarithromycin, 32% for metronidazole, and 11% for levofloxacin, with higher rates in southern Europe than in northern Europe [1].

In this scenario, Italy emerges as one of the European countries with the highest prevalence of *H. pylori* infection, where resistance to clarithromycin and levofloxacin is most frequently detected (36.9% and 29.2%, respectively) [13,24].

However, the prevalence of infection and antibiotic resistance vary widely from region to region, and studies published in the literature focus on local cases. In this review, we evaluate the antibiotic resistance rates of *H. pylori* in Italy with the aim of understanding and quantifying the actual *H. pylori* resistance status and identifying the optimal therapeutic approach.

### Genomic and Virulence Factors

*H. pylori* has several characteristics that explain its virulence and resistance to defense mechanisms. After invading the stomach, *H. pylori* neutralize the hostile acidic environment using its urease activity. This enzyme can neutralize hydrochloric acid by using nickel as a co-factor, which therefore is important for Helicobacter pathogenicity. The cell then moves to the gastric epithelium with the help of its flagella. *H. pylori* adhesins further interact with host cell receptors, resulting in successful colonization and persistent infection. After successful colonization, *H. pylori* produces several effector proteins/toxins that are responsible for host tissue damage. *H. pylori* forms biofilms that help reduce its susceptibility to antibiotics. Another resistance mechanism is the acquisition of a rod form under unfavorable environmental conditions (temperature or pH changes, long intervals between meals, and therapy with antibiotics or proton pump inhibitors) [25,26].

The best-studied virulence factors of *H. pylori* are the CagA protein, encoded in the cytotoxin-associated genes pathogenicity island (cagPAI), and the VacA protein (vacuolating cytotoxin A). The cagPAI consists of a 40 kb region containing up to 32 genes encoding components of a bacterial secretion system type IV (T4SS) involved in the translocation of an effector protein, CagA, into gastric epithelial cells. Upon translocation of this protein into host cells, CagA interacts with various host cell molecules, leading to an enhanced inflammatory response, hyperplasia of the gastric epithelium, and the formation of gastric polyps and adenocarcinomas. After incorporation into host cells, VacA accumulates in various cellular compartments and can induce apoptosis through loss of mitochondrial transmembrane potential, release of cytochrome C, and activation of the pro-apoptotic factor Bcl-2-associated X protein (Bax). VacA promotes *H. pylori* persistence by inhibiting T-cell proliferation and activation. It also triggers the inflammatory response by activating NF-kB and upregulating interleukin-8 (IL-8). VacA is associated with peptic ulcers and gastric cancer. Other virulence factors include integrative and conjugative elements (ICEs, genomic islands characterized by great plasticity and associated with severe gastric inflammation, apoptosis, and carcinogenesis), urease, structural and chemotactic flagellar proteins, and outer membrane proteins (OMPs). *H. pylori* encodes five families of OMPs. The outer membrane porins (Hop) or Hop-related proteins (Hor) are responsible for the transport of various molecules, including antibiotics, by passive diffusion. The adhesins SabA and BabA bind epithelial cells; the iron-regulated outer membrane proteins, which include the FecA- and FrpB-like proteins, bind heme and hemoglobin; the efflux pump outer membrane proteins, which expel various molecules from the bacterial cytoplasm; the Hof and Hom families, which are involved in adhesion to gastric cells [27,28].

The mechanisms of resistance to major antibiotics will be described in the next session.

## 2. Resistance Mechanisms of Principal Antibiotics Used for *H. pylori* Eradication

### 2.1. Clarithromycin 

**Figure 1:**
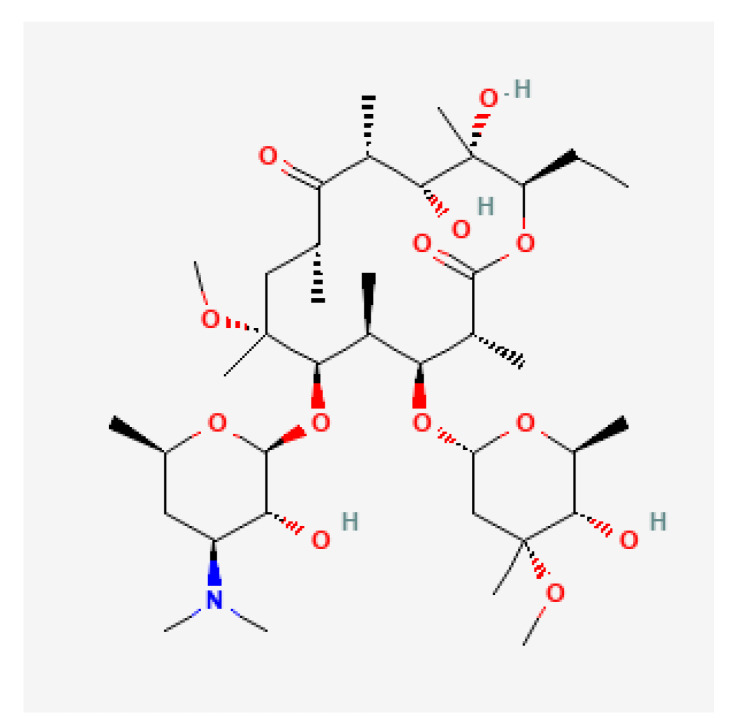
2D chemical structure of clarithromycin.

Clarithromycin (Figure 1) is a macrolide antibiotic that penetrates the cell wall of bacteria and reversibly binds to domain V of the 23S ribosomal RNA of the 50S subunit of the bacterial ribosome, blocking aminoacyl transfer RNA translocation and polypeptide synthesis. Its spectrum of activity includes many Gram-positive (*S. aureus*, *S. pneumoniae*, and *S. pyogenes*) and Gram-negative aerobic bacteria (*H. influenzae*, *H. parainfluenzae*, and *M. catarrhalis*), many anaerobic bacteria, *mycobacteria*, *Ureaplasma*, *Toxoplasma*, and *Borrelia spp*. Other aerobic bacteria against which clarithromycin is effective include *C. pneumoniae* and *M. pneumoniae* [29,30,31,32].

Clarithromycin resistance is due to point mutations in the peptidyltransferase region encoded in domain V of 23S rRNA, which reduces the binding ability of clarithromycin to the ribosomal subunit responsible for specific antibiotic-induced protein synthesis [33,34]. Three mutations in this domain, namely the A2141G/C and A2143G mutations are responsible for >90% of clarithromycin-resistant *H. pylori* strains [35]. Using next-generation sequencing technology, Binh et al. demonstrated that mutations in ribosomal protein L22p and translation initiation factor IF-2 also interact with 23S rRNA domains and play a key role in clarithromycin resistance [36]. Another mechanism involved in clarithromycin resistance is related to multidrug efflux pump transporters (MEPT). Of the five MEPT families, the resistance-nodulation-cell-division (RND) family is the most important in the development of clarithromycin resistance [37].

### 2.2. Levofloxacin

**Figure 2:**
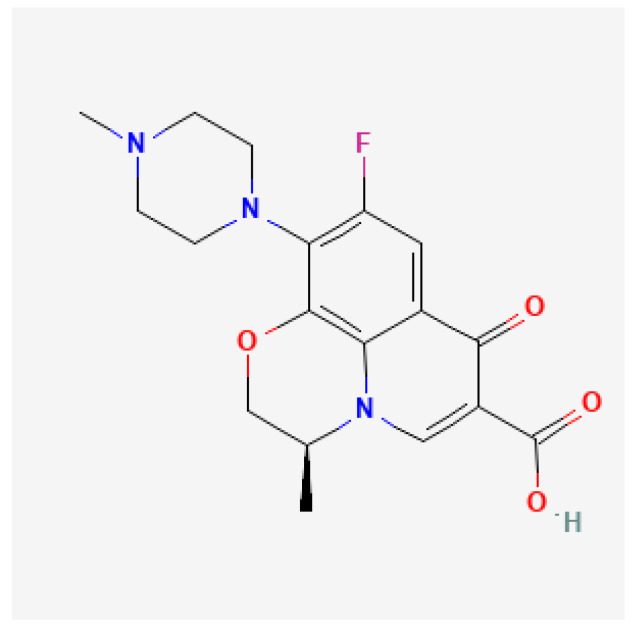
2D chemical structure of levofloxacin.

Levofloxacin (Figure 2), like other fluoroquinolone antibiotics, exerts its antimicrobial action by inhibiting two key bacterial enzymes: DNA gyrase and topoisomerase IV. DNA gyrase is an enzyme found only in bacteria that incorporates negative supercoils into DNA during replication. This helps to reduce the torsional stress caused by the introduction of positive supercoils during replication, and these negative supercoils are essential for chromosome condensation and the promotion of transcription initiation. The bacterial topoisomerase IV contributes to the relaxation of positive supercoils and is also important in the final stages of DNA replication to “unlink” newly replicated chromosomes to complete cell division. The result is a blockade of DNA replication that inhibits cell division and causes cell death. Levofloxacin has been shown to be effective in vitro against a range of aerobic Gram-positive and Gram-negative bacteria and may also be effective against certain genera of anaerobic bacteria such as *Chlamydia* spp. and *Legionella* spp. [29,38]. The A subunit is the major binding site for fluoroquinolones. Point mutations in the quinolone resistance-determining regions (QRDR) of the gyrA gene, particularly in the regions encoding amino acids 87 and 91, are the major determinants of fluoroquinolone resistance [39,40]. It has been demonstrated that a mutation at position 463 in *gyrB* may also be responsible for some cases of fluoroquinolone-resistant *H. pylori* [41].

### 2.3. Metronidazole

**Figure 3:**
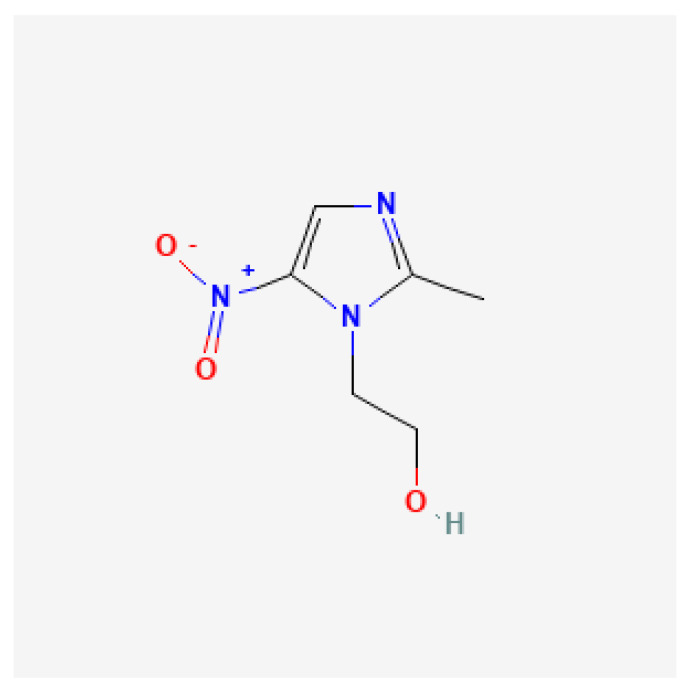
2D chemical structure of metronidazole.

Metronidazole (Figure 3) is a bactericidal antibiotic that belongs to the nitroimidazole group. Because it is a prodrug, metronidazole must undergo reductive activation of the nitro group by cellular electron acceptors. This reductive process, mediated primarily by oxy- gene-insensitive NADPH nitroreductase (RdxA), NADPH flavin oxidoreductase (FrxA), and ferredoxin-like enzymes (FrxB), results in the formation of nitro-anion-free radicals that directly damage subcellular structures and DNA. Metronidazole has shown antibacterial activity against most obligate anaerobes. However, in vitro studies, it did not show significant activity against facultative anaerobes or obligate aerobes. It is also used to treat amebiasis, trichomoniasis, and giardiasis [29,42,43]. In *H. pylori*, metronidazole resistance is mainly due to several mutations such as frameshift, insertion, and deletion involving genes encoding RdxA and, to a lesser extent, FrxA. Other suspected mechanisms of metronidazole resistance require mutations of enzymes involved in DNA repair and regulation of oxidative stress processes. These include the mutation of ferric uptake regulator (Fur), which leads to an abnormal increase in superoxide dismutase expression [44].

### 2.4. Amoxicillin

**Figure 4:**
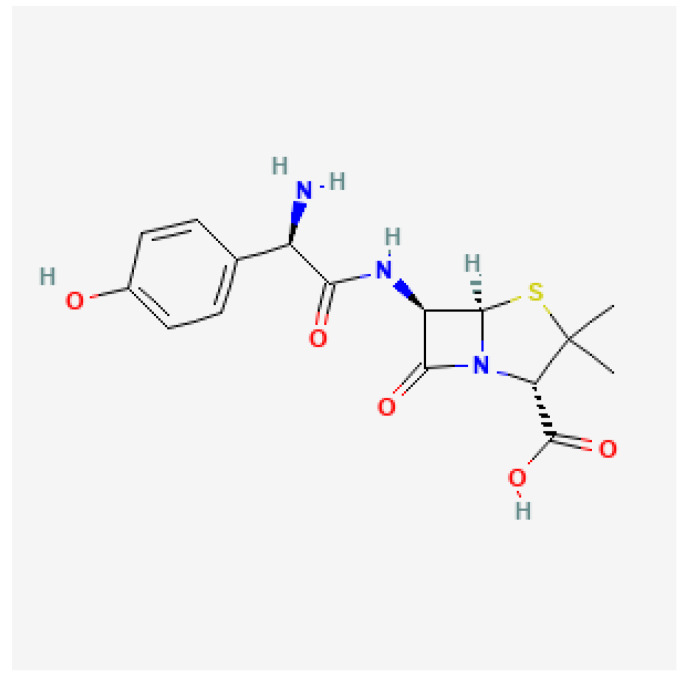
2D chemical structure of amoxicillin.

Amoxicillin (Figure 4) is a semisynthetic aminopenicillin antibiotic with broad-spectrum and bactericidal activity used mainly to treat infections of the respiratory tract, genitourinary system, and skin. Amoxicillin binds to and inactivates penicillin-binding proteins (PBPs) located on the inner membrane of the bacterial cell wall. Inactivation of PBPs impairs the cross-linking of peptidoglycan chains, which are required for the strength and rigidity of the bacterial cell wall. This disrupts bacterial cell wall synthesis, leading to weakening of the bacterial cell wall and cell lysis [29,45]. The major determinants of amoxicillin resistance in *H. pylori* are mutations in pbp1A, a gene encoding a specific PBP called PBP1A, resulting in decreased binding affinity to PBP1A. Other putative mechanisms involved in amoxicillin resistance include mutations in genes encoding efflux pumps and porins such as hofH, hefC, and hopC [46,47,48]. 

### 2.5. Tetracycline

**Figure 5:**
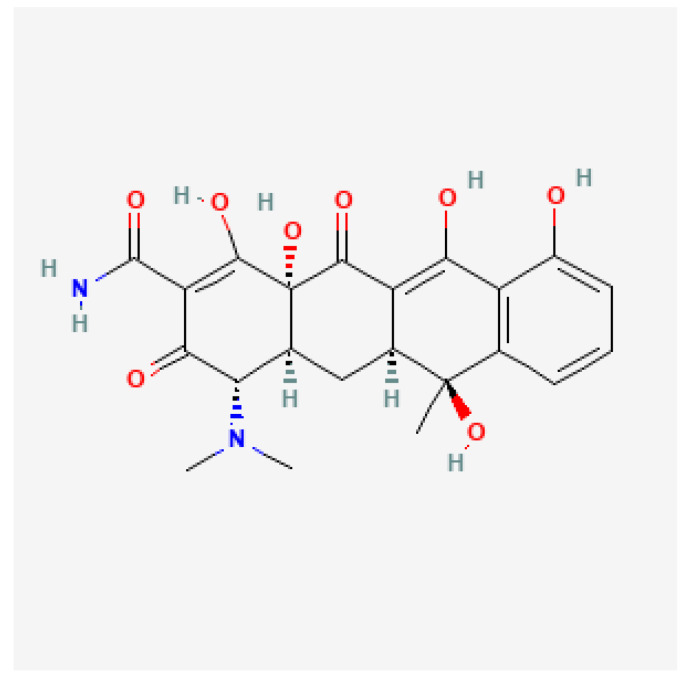
2D chemical structure of tetracycline.

Tetracyclines (Figure 5) are bacteriostatic antibiotics that bind reversibly to the 16SrRNA in the 30S subunit of bacterial ribosomes, preventing the binding of amino-acyl-tRNA to the A site of the ribosome and impairing protein synthesis and bacterial growth. They are used to treat bacterial infections such as Rocky Mountain spotted fever, typhoid fever, tick fever, Q fever, rickettsialpox, and Brill–Zinsser disease. May be used to treat infections caused by *Chlamydiae* spp., *B. burgdorferi* (Lyme disease), and upper respiratory tract infections caused by typical (*S. pneumoniae*, *H. influenzae*, and *M. catarrhalis*) and atypical organisms (*C. pneumoniae*, *M. pneumoniae*, *L. pneumophila*) [29,49,50]. In *H. pylori*, tetracycline resistance is mainly associated with single, double, or triple mutations in the 16S rRNA gene [51].

### 2.6. Rifabutin

**Figure 6:**
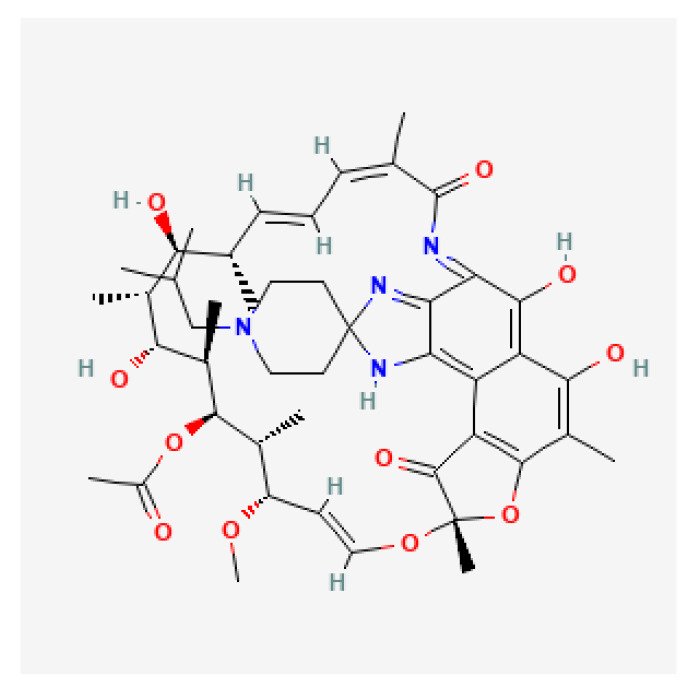
2D chemical structure of rifabutin.

Rifabutin (Figure 6), a member of the rifamycin group, is a bactericidal antibiotic that binds to the beta subunit of *H. pylori* DNA-dependent RNA polymerase encoded by the rpoB gene, thereby inhibiting bacterial transcription. Specifically, it interacts with bacterial RNA polymerase but does not inhibit the mammalian enzyme. It is bactericidal and has a very broad spectrum of activity against most Gram-positive and Gram-negative organisms (including *Pseudomonas aeruginosa*) and specifically *Mycobacterium tuberculosis* [29,52]. Point mutations in the rifampicin resistance determine region (RRDR) of the rpoB gene are responsible for resistance to rifabutin [39,53].

## 3. Methods for the Detection of *H. pylori* Resistance to Antibiotic 

Current European guidelines recommend antibiotic susceptibility testing even before prescribing first-line therapy, with respect to antibiotic stewardship [17].

Antibiotic resistance of *H. pylori* can be detected by phenotypic or genotypic methods. The former are culture-based methods that include gradient diffusion susceptibility testing (E-test), agar dilution, broth microdilution, and disc diffusion methods. These are the most commonly used tests to assess antibiotic susceptibility of *H. pylori*.

These methods allow quantitative determination of the minimum inhibitory concentrations (MIC) of antibiotics.

In the agar dilution method, antibiotic susceptibility is determined by growing *H. pylori* on agar plates with a twofold serial dilution of antibiotics [54].

Compared with other techniques, agar dilution is generally considered the method of choice for antibiotic resistance detection, but because it is technically more demanding and time consuming, it is not routinely used [55,56]. Disc diffusion is the simplest and least expensive method for routine susceptibility testing. In this method, antibiotic discs can be placed on an agar plate containing bacteria, and after incubation, the zone of inhibition is measured to determine antibiotic susceptibility. However, this method is not usually recommended for slow-growing bacteria such as *H. pylori* because unstable antibiotic patterns are released from the discs [56].

The E-test is a quantitative variant of the disc diffusion method with sensitivity and specificity of 45% and 98%, respectively [57]. It has been shown that there is a very good correlation between agar dilution results and the MIC of the E-test for most antibiotics, with the exception of metronidazole (the E-test may overestimate the resistance rate of metronidazole by 10–20%). The European Committee on Antimicrobial Susceptibility Testing (EUCAST) recommends the E-test and corresponding MIC breakpoints for susceptibility testing to *H. pylori* for six antibiotics (levofloxacin, clarithromycin, metronidazole, amoxicillin, tetracycline, and rifampicin) [58].

Although phenotypic testing is the conventional method for assessing antibiotic susceptibility, it is time consuming, complex, and unsuccessful about 10% of the time because the bioptic sample may be contaminated or the bacteria may not grow. Because of these problems, molecular methods offer an attractive alternative for determining antibiotic resistance. These methods allow detection of point mutations in the bacterial genome that are responsible for antibiotic resistance [59].

Currently, nucleic acid-based tests are available for the detection of clarithromycin, tetracycline, and fluoroquinolone resistance [54]. In contrast, molecular methods have not yet been developed for the detection of amoxicillin, and metronidazole. Rifabutin resistance can only be determined by sequencing [60].

Molecular techniques include dual-priming oligonucleotide (DPO) multiplex PCR, PCR-restriction fragment length polymorphism (RFLP), real-time PCR, PCR-DNA enzyme immunoassay, mismatched PCR, hybridization, fluorescence in situ hybridization (FISH), and sequencing techniques [59]. They detect the presence of antibiotic resistance in *H. pylori* from biopsy specimens, gastric fluid, colonies, and stool samples [61,62]. PCR methods based on the detection of point mutations have a sensitivity of 98% and a specificity of 92%.

## 4. Clinical Determinants of Antibiotic Resistance

Several clinical factors have been associated with antibiotic-resistant *H. pylori*, including previous antibiotic exposure, older age, female sex, geographic region, ethnicity, alcohol consumption, and non-ulcer dyspepsia [63]. However, the most important risk factor associated with antibiotic resistance is previous antibiotic use [64]. Antibiotics exert strong selection pressure on bacteria, leading to strain mutations and thus antibiotic resistance. *H. pylori* includes strains with hypermutability that develop high-frequency mutations, which may be the reason for the rapid emergence of resistance after antibiotic treatment [65]. Lim et al. demonstrated in a cohort study that prior exposure to clarithromycin and other macrolides was an independent risk factor for failure of first-line triple therapy based on AMX and CLR [64]. In a European multicenter survey on prior antibiotic use and antibiotic resistance, outpatient use of antibiotics such as CLR and quinolones prior to first-line treatment for *H. pylori* eradication correlated positively with *H. pylori* antibiotic resistance [66]. In a study of 2063 *H. pylori*-positive patients, McNulty et al. demonstrated that prior treatment with clarithromycin, levofloxacin, and metronidazole increased the risk of resistance to these antibiotics [67]. For this reason, patients who do not respond to *H. pylori* treatment with clarithromycin, fluoroquinolones, or metronidazole should not be retreated with these antibiotics. Currently, there are no strong data to support that previous treatment with bismuth, tetracycline, and amoxicillin increases the risk of antibiotic resistance in *H. pylori*. To date, guidelines allow retreatment with these drugs [18]. Geographic location can affect antibiotic resistance rates, which may be due to local patterns of use of certain antibiotics in the community as well as other factors related to healthcare systems [1]. A multicenter observational study conducted in Europe by Megraud et al. showed that the region in which patients were born was significantly associated with clarithromycin, levofloxacin, and metronidazole resistance in multivariable analyses. In fact, patients born in Southern Europe or Western/Central Europe had a higher percentage of clarithromycin and levofloxacin resistance than patients born in Northern Europe [13].

Furthermore, antibiotic resistance to *H. pylori* can vary between ethnic groups even in the same geographic area, suggesting differences in access to healthcare and the presence of genetic and cultural factors [63].

Data from several studies demonstrate increased levofloxacin resistance in elderly patients with *H. pylori*, whereas age does not appear to play a role in clarithromycin and metronidazole resistance [13,68,69]. Children have lower rates of *H. pylori* antibiotic resistance than adults, as reported by Savoldi et al. in a meta-analysis [1]. On the other hand, higher rates of resistance to clarithromycin, metronidazole, and levofloxacin were found in women. Although the exact mechanism is not yet clear, this may be metronidazole is mainly used to treat gynecological infections and quinolones to treat low urinary tract infections [63].

In addition, alcohol consumption may play a role in *H. pylori* antibiotic resistance. Indeed, several studies have shown that increased metronidazole resistance is associated with lower alcohol consumption. Because co-administration of metronidazole and alcohol produces a disulfiram-like response, the propensity to prescribe this antibiotic may be lower in patients who consume alcohol, and this fact may prevent the development of metronidazole resistance. However, no studies have shown that alcohol consumption promotes *H. pylori* eradication [69,70].

Several studies have shown that patients with gastroduodenal ulcers achieve a higher eradication rate of *H. pylori* than patients with non-ulcer dyspepsia [64,71]. In addition, resistance to clarithromycin has been observed more frequently in patients without peptic ulcers [68,72,73]. Patients with gastric or duodenal ulcers have a higher rate of cag-A-positive strains, which promote the development of gastrointestinal damage. On the other hand, cagA-positive strains are more sensitive to CRL [74].

## 5. Empiric vs. Tailored Therapy

### 5.1. First Line Therapy 

Several studies have compared the cure rates of empiric therapy with those of susceptibility test-guided therapy (SGT) in the initial treatment of *H. pylori* infection [75]. In their meta-analysis, Wenzhen et al., Lopez-Gongora et al., and Chen et al. concluded that SGT was more effective than empiric therapy in initial treatment. However, it should be clarified that in these meta-analyses most first-line therapies were based on clarithromycin, so the data cannot be generalized to treatments based on other antibiotics (e.g., bismuth and metronidazole) [76,77,78]. Another recent (2021) meta-analysis by. Gingold-Belfer et al. compared empiric treatment of *H. pylori* with SGT. They found that SGT was slightly more effective than first-line empiric clarithromycin triple therapy only when clarithromycin resistance exceeded 20%. In contrast, susceptibility-guided therapy was not superior to empiric therapy when quadruple therapy was used as first-line therapy [79].

### 5.2. Second-Line Therapy 

Lopez-Gongora et al. performed a meta-analysis including four RCTs comparing SGT with empiric treatment as second-line therapy and found no significant differences between the two strategies. The same results were reported by Chen et al. Baylina et al. too found that susceptibility-guided treatment alone did not achieve adequate cure rates for second line therapies [76,77,80]. 

### 5.3. Third-Line Therapy

European guidelines state that after failure of two antibiotic regimens, a susceptibility test is recommended to guide treatment. In the meta-analysis mentioned earlier, there was no RCT comparing cure rates of sensitivity-guided therapy with empiric third-line treatment [77,78,80].

A recent systematic review by Puig et al. evaluated the efficacy of SGT in the treatment of *H. pylori* after two failed trials. Four noncomparative observational studies were included and found a median cure rate of 72% with sensitivity-guided treatment. Based on these results, the authors concluded that SGT may be an acceptable alternative for rescue treatment, albeit with suboptimal cure rates [81].

## 6. Alternative Therapies for *H. pylori*

### 6.1. Probiotics

Probiotics are defined as live microorganisms that, when administered in sufficient quantity, provide health benefits to the host [82]. They consist of microorganisms belonging to the group of bacteria or yeasts. The two species most commonly used as probiotics in clinical practice are lactic acid-producing bacteria such as lactobacilli and bifidobacteria. 

Most probiotics colonize the human intestine, and some of them, such as *Lactobacillus* spp., colonize the human stomach and act directly or indirectly against *H. pylori* [83].

Due to increasing antibiotic resistance and the occurrence of side effects, which are the most common cause of treatment failure against *H. pylori*, probiotics are gaining interest as an adjunct to standard antibiotics. Published studies suggest that probiotics can increase eradication rates and decrease gastrointestinal side effects caused by antibiotics [84,85,86,87]. Probiotics may act through several direct and indirect mechanisms, including the secretion of antibacterial substances, inhibition of bacterial adhesion, improvement of the mucosal barrier, and immunomodulation [88,89,90].

Probiotics can inhibit the growth of *H. pylori* by secreting antibacterial substances such as short-chain fatty acids (SCFAs), lactic acid, hydrogen peroxide, and bacteriocins. Lactic acid produced by probiotics during carbohydrate metabolism can also exert its antimicrobial effects by lowering pH and inhibiting urease activity of *H. pylori* [91]. Bhatia et al. observed for the first time an antagonistic effect of a Lactobacillus strain against H. pylori due to SCFAs [92]. Certain Lactobacillus species can produce bacteriocins, thermostable peptides with antagonistic activity against biofilm cells, and some of them have shown potent high-frequency antibacterial activity against *H. pylori* in vitro [90].

Probiotics can strengthen the mucosal barrier to prevent *H. pylori* colonization. *H. pylori* can suppress the expression of the muc1 and muc5A genes, resulting in disruption of the protective mucin layer on the surface. In vitro studies have shown that probiotics such as L. plantarum and L. rhamnosus increase the expression of the MUC2 and MUC3 genes and increase the extracellular secretion of mucin by colon cell cultures, thereby restoring gastric mucosal permeability and inhibiting the adherence of pathogenic bacteria such as *H. pylori* [93].

In terms of immunological mechanisms, probiotics have been shown to modulate the host immunological response by acting on the secretion of anti-inflammatory cytokines, leading to a reduction in gastric activity and inflammation [12]. Oh et al. investigated the effect of probiotics in addition to antibiotics on the gut microbiota during treatment for *H. pylori* eradication in a randomized controlled trial. They showed that probiotics can limit the growth of resistant bacteria by reducing the imbalance in the composition of the gut microbiota [94]. Although several systematic reviews and meta-analyses of RCTs suggest that the use of probiotics in combination with antimicrobial therapy may increase eradication rates and decrease adverse effects, the role of probiotics in eradicating this infection is not well defined, and the overall evidence for a beneficial effect is low [95,96]. The Toronto guidelines discourage the routine use of probiotics to reduce adverse events or increase eradication rates [19]. The American College of Gastroenterology states that the use of probiotics may be promising, but that uncertainties about the optimal dose, timing of administration, and duration of therapy should be resolved before recommending their use in clinical practice [18]. The European guidelines state that only some types of probiotics (including different strains of *Lactobacillus* spp., *Bifidobacterium* spp., and *Saccharomyces boulardii*) have been shown to reduce adverse events, and therefore these probiotics should be selected on a case-by-case basis as adjunctive therapy to reduce adverse events [17].

### 6.2. Vonoprazan 

Vonoprazan is a new potassium-competitive acid blocker (PCAB) with a faster onset of action and deeper and more sustained suppression of gastric acid secretion than PPIs. Because vonoprazan lacks anti-*H. pylori* activity in vitro, its high eradication rate is closely related to its potent inhibition of gastric acid secretion [97]. Vonoprazan has been clinically available in Japan since 2015, where it is currently approved for first-line *H. pylori* eradication with clarithromycin-containing triple therapy and for second-line therapy with metronidazole and amoxicillin [98]. While an RCT found no significant differences between lansoprazole- and vonoprazan-containing CLR triple therapy in the treatment of clarithromycin-susceptible *H. pylori* strains, CLR triple therapy with vonoprazan is superior to conventional PPI-based therapy for CLR-resistant strains [98,99,100]. Vonoprazan is mainly used in Japan, where clarithromycin triple therapy is still considered first-line therapy despite *H. pylori* resistance exceeding 30%. Further studies are needed to investigate and validate the applicability of vonoprazan in other eradication regimens.

### 6.3. Vaccine

A vaccine could be a promising alternative for *H. pylori* eradication, but its development remains a major challenge [101]. Several bacterial antigens such as urease, catalase, CagA, VacA, BabA, HspA, the FliD protein, and multivalent epitopes have been considered for vaccine production. 

Although preclinical studies in mouse models have shown promising results for both preventive and therapeutic strategies, previous clinical trials have been unsuccessful, and most of the vaccines currently under investigation are still in preclinical stages or in phase-I trials [102,103,104].

A phase 3 randomized controlled trial conducted by Zeng et al. in China has demonstrated efficacy, safety, and immunogenicity of a three-dose oral recombinant urease B vaccine in *H. pylori*-infected children aged 6 to 15 years. Efficacy against natural *H. pylori* infection was 71.8%, and overall protection lasted up to three years [105]. Although this study demonstrated that a vaccine could protect against natural *H. pylori* infection, production of the vaccine was discontinued [106]. Further studies failed to achieve effective immunity [98,107]. A better understanding of both the infection mechanisms of *H. pylori* and the immune responses elicited by natural *H. pylori* infection is needed for the development of a vaccine for humans [106].

## 7. Rates of Antibiotic Resistance in Italy 

### 7.1. Primary Resistance (Table 1, Table 2, Table 3 and Table 4)

Saracino et al. analyzed 1763 *H. pylori*-positive patients between 2009 and 2014, 907 of whom received no previous treatment and had an antibiogram. They compared resistance rates to the most used antibiotics with a cohort of 1415 patients diagnosed with *H. pylori* infection between 2015 and 2019 (739 untreated patients in this group had an antibiotic susceptibility test). Primary *H. pylori* antibiotic resistance rates in the first and second five-year periods were 30.2% and 37.8%, respectively, for clarithromycin, 25.6% and 33.8%, respectively, for levofloxacin (resistance rates for both antibiotics increased significantly in the subsequent five years), and 33.3% and 33.6%, respectively, for metronidazole (no significant increase) [108].

Fiorini et al. analyzed antibiotic susceptibility data from 1424 *H. pylori* strains of treatment-naïve patients between 2010 and 2016. Resistance to clarithromycin showed an increasing trend from 2010 to 2013 (from 19% to 35.6%) and reached a plateau in 2016 (35.9%). Resistance to levofloxacin also increased between 2010 and 2013 (from 19% to 29.7%) and reached a plateau in 2016 (29.3%), as did resistance to metronidazole (from 33.6% in 2010 to 45.3% in 2013), which reached a plateau in 2016 (40.2%) [109].

Gatta et al. studied 1682 treatment-naive *H. pylori*-positive patients between 2010 and 2015 and found an overall primary resistance rate of 36.1% to clarithromycin, 28.7% to levofloxacin, and 38.6% to metronidazole, with all showing an increasing trend from 2010 to 2015 [110].

Palmitessa et al. analyzed antibiotic resistance in 92 *H. pylori* strains from patients (both treatment-naive and previously treated) between 2017 and 2018, using phenotypic and genotypic methods (PCR on *H. pylori* isolates from cultures) for clarithromycin and levofloxacin. They found a primary resistance rate to clarithromycin of 37.7%, with concordance between the phenotypic and genotypic assays. The primary resistance rate to levofloxacin was 26.2%, with concordance also found for this antibiotic. The primary resistance rates for metronidazole, amoxicillin, tetracycline, and rifabutin were 16.4%, 1.6%, 0%, and 1.6%, respectively [111].

Losurdo et al. recruited both treatment-naive and non-naive patients from January 2017 to July 2020 and collected a stool sample, which was analyzed at RT-PCR to detect point mutations conferring resistance to clarithromycin and levofloxacin to *H. pylori*. Among the 135 treatment-naive patients included, the primary resistance rate to clarithromycin was 27.4%, with no significant change in the time trend from 2017 to 2020 (resistance rates ranged from 30% in 2017 to 22.2% in 2020). The overall primary resistance rate to levofloxacin was 19.2, with a dramatic increase in rates from 2017 (10%) to 2018 (3.3%), 2019 (20%), and 2020 (37.8%) (*p* = 0.001) [112].

**Table 1:** Primary clarithromycin resistance in Italy.

Method	Year	Resistance %	Region	Research Group	Ref.
Culture on biopsy	2020	30.2% (95% CI 27.2–33.3) in 2009–2014, 37.8% (95% CI 34.2–41.4) in 2015–2019	Emilia-Romagna	Saracino et al.	[108]
Culture on biopsy	2018	19% in 2010, 35.6% in 2013, 35.9% in 2016 (OR not calculated)	Emilia-Romagna	Fiorini et al.	[109]
Culture on biopsy	2018	36.1% in 2010–2015	Emilia-Romagna	Gatta et al.	[110]
Culture on biopsy-PCR on isolates	2020	37.7% in 2017–2018	Puglia	Palmitessa et al.	[111]
RT-PCR on stools	2020	27.4% in 2017–2020	Puglia	Losurdo et al.	[112]

Abbreviations: CI, confidence interval; PCR, polymerase chain reaction; RT, real-time.

**Table 2:** Primary levofloxacin resistance in Italy.

Method	Year	Resistance %	Region	Research Group	Ref.
Culture on biopsy	2020	25.6% in 2010–2014 and 33.8% in 2015–2019	Emilia- Romagna	Saracino et al.	[108]
Culture on biopsy	2018	19% in 2019, 29.7% in 2013, 29.3% in 2016 (OR not calculated)	Emilia- Romagna	Fiorini et al.	[109]
Culture on biopsy	2018	28.7% in 2010–2015	Emilia- Romagna	Gatta et al.	[110]
Culture on biopsy-PCR on isolates	2020	26.2 % in 2017–2018	Puglia	Palmitessa et al.	[111]
RT-PCR on stools	2020	19.2 % in 2017–2020	Puglia	Losurdo et al.	[112]

Abbreviations: PCR, polymerase chain reaction; RT, real-time.

**Table 3:** Primary metronidazole resistance in Italy.

Method	Year	Resistance %	Region	Research Group	Ref.
Culture on biopsy	2020	33.3 % in 2010–2014 and 33.6%in 2015–2019	Emilia- Romagna	Saracino et al.	[108]
Culture on biopsy	2018	33.6 % in 2019, 45.3% in 2013, 40.2% in 2016 (OR not calculated)	Emilia- Romagna	Fiorini et al.	[109]
Culture on biopsy	2018	38.6% in 2010–2015	Emilia- Romagna	Gatta et al.	[110]
Culture on biopsy	2020	16.4 % in 2017–2018	Puglia	Palmitessa et al.	[111]

**Table 4:** Primary resistance to the other antibiotics in Italy.

Antibiotic	Method	Year	Resistance %	Region	Research Group	Ref.
Amoxicillin	Culture on biopsy	2020	1.6 % in 2017–2018	Puglia	Palmitessa et al.	[111]
Tetracyclin	Culture on biopsy	2020	0 % in 2017–2018	Puglia	Palmitessa et al.	[111]
Rifabutin	Culture on biopsy	2020	1.6 % in 2017–2018	Puglia	Palmitessa et al.	[111]

### 7.2. Secondary Resistance (Table 5, Table 6, Table 7 and Table 8)

Saracino et al. analyzed antibiotic susceptibility testing of 1037 *H. pylori* strains from patients who had failed at least one eradication therapy. Overall resistance rates were 83.1% for clarithromycin, 47.2% for levofloxacin, and 66.7% for metronidazole. Resistance rates increased for all drugs depending on the number of prior treatment attempts [21].

In 2018, Mascellino et al. analyzed data from 40 patients infected with *H. pylori* who had been previously treated with one or more eradication attempts. Among culture-positive patients, they found resistance rates to clarithromycin in 50%, to levofloxacin in 25%, to metronidazole in 68%, to amoxicillin in 4%, and to tetracycline in 6% of cases [113].

The same group retrospectively studied a group of 80 patients with upper gastritis who had failed prior first-line therapy, had a positive urea breath test (UBT), and had upper endoscopy. They investigated the secondary resistance of *H. pylori* to the most common antibiotics by examining both phenotypic susceptibility (MIC results) of *H. pylori* in cultures and genotypic susceptibility to clarithromycin and levofloxacin from gastric biopsies using molecular methods. They found a clarithromycin resistance rate of 35% by phenotypic methods and 42.5% by genotypic methods (the difference was not statistically significant) and a levofloxacin resistance rate of 15% by phenotypic methods and 30% by genotypic methods (the difference was statistically significant). These results suggest that genotypic methods are more sensitive than phenotypic methods in detecting resistant *H. pylori* strains. Resistance rates for metronidazole, amoxicillin, and tetracycline were 61.6%, 1.25%, and 2.5%, respectively [114].

As mentioned earlier, Palmitessa et al. analyzed 92 patients infected with *H. pylori* and found secondary resistance rates of 83.9% for clarithromycin, 64.5% for levofloxacin, 64.5% for metronidazole, and 6.5%, 0%, and 0% for amoxicillin, tetracycline, and rifabutin, respectively [111].

Losurdo et al. also recruited 91 patients with at least one failed eradication; 56 had single failure on a clarithromycin-containing regimen, whereas 35 experienced double failure on both a clarithromycin-containing regimen and levofloxacin-based triple therapy. The resistance rate to clarithromycin was 64.8% with a stable time trend from 2017 to 202 (*p* = 0.85) and to levofloxacin was 59.3%, with the highest rate observed in 2019 (68.4%) and the lowest in 2018 (51.6%), with no significant trend [112].

**Table 5:** Secondary clarithromycin resistance in Italy.

Method	Year	Resistance %	Region	Research Group	Ref.
Culture on biopsy	2020	83.1% in 2009–2019	Emilia-Romagna	Saracino et al.	[21]
Culture on biopsy-PCR on isolates	2020	83.9% in 2017–2018	Puglia	Palmitessa et al.	[111]
RT-PCR on stools	2020	64.8% in 2017–2020	Puglia	Losurdo et al.	[112]
Culture on biopsy-RT-PCR on isolates	2018	50% (not indicated the years)	Lazio	Mascellino et al.	[113]
Culture on biopsy-RT-PCR on isolates	2020	35% with phenotypic methods, 42.5% with genotypic methods (not indicated the years)	Lazio	Mascellino et al.	[114]

Abbreviations: PCR, polymerase chain reaction; RT, real-time.

**Table 6:** Secondary levofloxacin resistance in Italy.

Method	Year	Resistance %	Region	Research Group	Ref.
Culture on biopsy	2020	47.2% in 2009–2019	Emilia-Romagna	Saracino et al.	[21]
Culture on biopsy-PCR on isolates	2020	64.5% in 2017–2018	Puglia	Palmitessa et al.	[111]
RT-PCR on stools	2020	59.3% in 2017–2020	Puglia	Losurdo et al.	[112]
Culture on biopsy-RT-PCR on isolates	2018	25% (not indicated the years)	Lazio	Mascellino et al.	[113]
Culture on biopsy-RT-PCR on isolates	2020	15% with phenotypic methods, 30% with genotypic methods (not indicated the years)	Lazio	Mascellino et al.	[114]

Abbreviations: PCR, polymerase chain reaction; RT, real-time.

**Table 7:** Secondary metronidazole resistance in Italy.

Method	Year	Resistance %	Region	Research Group	Ref.
Culture on biopsy	2020	66.7% in 2009–2019	Emilia- Romagna	Saracino et al.	[21]
Culture on biopsy-PCR on isolates	2020	64.5% in 2017–2018	Puglia	Palmitessa et al.	[111]
Culture on biopsy-RT-PCR on isolates	2018	68% (not indicated the years)	Lazio	Mascellino et al.	[113]
Culture on biopsy-RT-PCR on isolates	2020	61.6% (not indicated the years)	Lazio	Mascellino et al.	[114]

Abbreviations: PCR, polymerase chain reaction; RT, real-time.

**Table 8:** Secondary resistance to the other antibiotics in Italy.

Antibiotic	Method	Year	Resistance %	Region	Research Group	Ref.
Amoxicillin	Culture on biopsy	2020	6.5% in 2017–2018	Puglia	Palmitessa et al.	[111]
Rifabutin	Culture on biopsy	2020	0% in 2017–2018	Puglia	Palmitessa et al.	[111]
Amoxicillin	Culture on biopsy-RT-PCR on isolates	2018	4% (not indicated the years)	Lazio	Mascellino et al.	[113]
Amoxicillin	Culture on biopsy-RT-PCR on isolates	2020	1.25% (not indicated the years)	Lazio	Mascellino et al.	[114]
Tetracycline	Culture on biopsy	2020	0% in 2017–2018	Puglia	Palmitessa et al.	[111]
Tetracycline	Culture on biopsy-RT-PCR on isolates	2018	6% (not indicated the years)	Lazio	Mascellino et al.	[113]
Tetracycline	Culture on biopsy-RT-PCR on isolates	2020	2.5% (not indicated the years)	Lazio	Mascellino et al.	[114]

Abbreviations: PCR, polymerase chain reaction; RT, real-time.

## 8. Eradication Rates with the Most Important Antibiotic Regimens

### First Attempt (Table 9 and Table 10)

Saracino et al. analyzed not only resistance rates to CLR, levofloxacin, and metronidazole but also eradication rates of sequential therapy in a cohort of therapy-naive *H. pylori*-infected individuals. The overall eradication rate of classical sequential therapy (5 days of dual therapy with 40 mg PPI twice daily and 1000 mg amoxicillin twice daily, followed by 5 days of triple therapy with 40 mg PPI twice daily and clarithromycin 500 mg plus metronidazole 500 mg both twice daily) was still optimal in this group and consistently exceeded 90%. The eradication rate was suboptimal (per protocol—PP—83.6%, intention to treat—ITT—77%) only in patients whose strains were resistant to both clarithromycin and metronidazole Eradication rates decreased significantly in the second five-year period, from PP 95.3% to PP 90.4% [108].

In a post hoc evaluation of a treatment trial in which consecutive patients with dyspeptic symptoms underwent upper endoscopy at a single center, De Francesco et al. analyzed data from 1006 treatment-naive patients. They related MIC values for clarithromycin and metronidazole to the eradication rate of sequential therapy. They classified the degree of antibiotic resistance based on MIC values as low (MIC of 0.5–8 for clarithromycin and of 8–32 for metronidazole) and high (MIC of 8–256 for clarithromycin and of 32–256 mg/L for metronidazole). The cure rates of sequential therapy in patients with *H. pylori* strains resistant to either clarithromycin or metronidazole did not differ significantly between subgroups with low or high MIC values of resistance. In patients with *H. pylori* strains exhibiting dual antibiotic resistance, the eradication rate (90.9%) was not significantly (*p* = 0.064) reduced in subjects with a low clarithromycin resistance compared with subjects with susceptible strains (95.8%). On the contrary, the cure rate (84.5%) was significantly lower (*p* = 0.0001) when the MIC of clarithromycin was high compared with the eradication rate in susceptible strains. These data suggest that bacterial resistance becomes relevant in vivo when strains with dual clarithromycin and metronidazole resistance have high MIC values for at least one of these antibiotics [115].

Gatta et al. analyzed resistance rates to clarithromycin, metronidazole, and levofloxacin and eradication rates of sequential therapy in 1120 therapy-naive patients. The overall eradication rates in the ITT and PP analyses were 91.1% and 93.7%, respectively. When sensitivity to clarithromycin and metronidazole was taken into account, the eradication rate in patients with strains sensitive to both antibiotics was 97.3%, whereas it was 93.4% in patients with strains resistant to clarithromycin but sensitive to metronidazole. Similarly, the eradication rate was 96.1% in patients with strains resistant to metronidazole but sensitive to clarithromycin. In contrast, only 83.1% of patients with strains resistant to both clarithromycin and metronidazole were eradicated. Finally, intermediate clarithromycin resistance (MIC between 0.25 mcg/mL and 0.50 mcg/mL) did not negatively affect eradication rates with sequential therapy. The authors concluded that sequential therapy works as well as concurrent therapy in the presence of resistance to one or two antibiotics [110].

Di Ciaula et al. analyzed 651 patients infected with *H. pylori* who underwent various eradication treatments. One hundred and ninety-one of them were therapy-naive, received clarithromycin-based sequential therapy, and achieved an eradication rate of 89.0% in the ITT and 89.9% in the PP analysis, whereas 92 of them (also therapy-naive) received standard triple therapy (PPI + clarithromycin 500 mg + amoxicillin 1000 mg, each administered twice daily for 7 days) and achieved an eradication rate of 70.7%. Patients reported abdominal bloating and flatulence as the main side effect of these therapies, but this did not affect their compliance. They also administered Pylera^®^-based therapy (bismuth subcitrate potassium 140 mg, metronidazole 125 mg, and tetracycline 125, administered four times daily) plus omeprazole 20 mg bid to 85 therapy-naïve patients achieving an eradication rate of 100% [116].

Romano et al. administered 10- or 14-day N-BQT (esomeprazole 40 mg, clarithromycin 500 mg, amoxicillin, and tinidazole 500 mg twice daily) to *H. pylori* treatment-naive patients not previously treated with clarithromycin. Patients who reported or had doubts about prior exposure to this antibiotic received a 10-day BQT (esomeprazole 40 mg twice daily and Pylera^®^ four times daily). Two hundred and three patients not previously treated with clarithromycin received N-BQT and achieved an eradication rate of 88.2% in the ITT and 91.2% in the PP analysis. The 201 patients previously treated with clarithromycin received BQT and achieved an eradication rate of 91.2% in the ITT and 95.8% in the PP analysis. Subgroup analysis showed that eradication rates were significantly higher with 14-day N-BQT than with 10-day N-BQT in both the ITT (96.1% vs. 80%, *p* = 0.001) and PP analyses (97% vs. 85.1%, *p* = 0.003). The efficacy of BQT was comparable to that of 14-day N-BQT (ITT: *p* = 0.137, PP: *p* = 0.614) and higher than that of 10-day N-BQT (ITT: *p* = 0.004, PP: *p* = 0.001). Compliance was good in 95.6% of patients in the N-BQT group and in 95% of patients in the BQT group. Treatment-related adverse events (TRAEs) were reported by 24.1% of patients in the first group and by 26.9% in the second group. The most common TRAEs were nausea, diarrhea, abdominal pain, dyspepsia, and vomiting in both treatment regimens. Most cases of TRAEs were mild or moderate; severe TRAEs leading to treatment discontinuation were reported by 9 patients (4.4%) in the N-BQT and by 10 patients in the BQT. The rates of adverse events and adverse events leading to treatment discontinuation were not statistically different between the two groups. The data suggest that administration of a clarithromycin-containing regimen without bismuth is effective and safe even in an area with a high prevalence of clarithromycin-resistant *H. pylori* strains when prior clarithromycin exposure is known. In addition, the efficacy of a bismuth-containing regimen has been confirmed in therapy-naive patients with prior clarithromycin exposure [117].

In a prospective study, Fiorini et al. compared the eradication rates of classical sequential therapy and bismuth-based therapy with esomeprazole 20 mg twice daily and Pylera three tablets four times daily for 10 days in a cohort of 495 treatment-naïve patients with *H. pylori* infection. After sequential (250 patients) and quadruple (245 patients) therapy, eradication rates were 92 and 91%, respectively, by ITT analysis and 96 and 97%, respectively, by PP analysis. Overall, the pattern of bacterial resistance did not significantly affect cure rates, but the presence of clarithromycin and metronidazole dual resistance tended to decrease the success rate of both sequential (84.8 vs. 90.1%; *p* = 0.4) and quadruple therapy (85 vs. 94.1%; *p* = 0.06). Adverse events, mainly gastrointestinal symptoms, headache, and dizziness, occurred more frequently with quadruple therapy than with sequential therapy (56.9 vs. 25.8%; *p* = 0.001), with two (0.9%) patients discontinuing treatment because of dizziness (n = 1) and vomiting (n = 1) with bismuth-based therapy, whereas none of the patients receiving the sequential regimen discontinued treatment because of adverse events. With this study, the authors demonstrated that in Italy, a country with high rates of resistance to CLR, sequential and bismuth-based quadruple therapy achieved similar eradication rates of *H. pylori* infection in first-line treatment [118].

**Table 9:** Eradication Rate of clarithromycin-based regimens in treatment naïve patients.

Antibiotic Regimen	Year	Eradication Rate %	Region	Research Group	Ref.
Clarithromycin-based sequential	2020	87.5% at ITT analysis, 93.4% at PP analysis,	Emilia- Romagna	Saracino et al.	[108]
Clarithromycin-based sequential	2018	91.1% at ITT analysis, 93.7% at PP analysis,	Emilia- Romagna	Gatta et al.	[110]
Clarithromycin-based sequential	2017	89% at ITT analysis, 89.9% at PP analysis	Puglia	Di Ciaula et al.	[116]
Standard Triple Therapy	2017	70.7%	Puglia	Di Ciaula et al.	[116]
Clarithromycin-based concomitant	2017	88.2% at ITT analysis, 91.2% at PP analysis,	Campania	Romano et al.	[117]
Clarithromycin-based sequential	2018	92% at ITT analysis, 96% at PP analysis,	Emilia- Romagna	Fiorini et al.	[118]

Abbreviations: ITT, Intention to Treat; PP, Per Protocol.

**Table 10:** Eradication Rate of bismuth-based regimens in treatment naïve patients.

Antibiotic Regimen	Year	Eradication Rate %	Region	Research Group	Ref.
Bismuth-based quadruple therapy (Pylera^®^)	2017	100%,	Puglia	Di Ciaula et al.	[116]
Bismuth-based quadruple therapy Pylera^®^)	2017	91.2% at ITT analysis, 95.8% at PP analysis	Campania	Romano et al.	[117]
Bismuth-based quadruple therapy (Pylera^®^)	2018	91% at ITT analysis, 97% at PP analysis	Emilia-Romagna	Fiorini et al.	[118]

Abbreviations: ITT, Intention to Treat; PP, Per Protocol.

## 9. Sequent Attempt (Table 11 and Table 12)

Saracino et al. also analyzed the eradication rates of the main therapeutic regimens for the treatment of *H. pylori* infections administered after antibiotic susceptibility testing. Overall cure rates in the ITT and PP analyzes were 84.2% and 91.4% for sequential therapy, 86.8% and 90% for Pylera^®^ plus esomeprazole 20 mg twice daily, 80.4% and 86.6% for levofloxacin-based therapy, and 75.6% and 83.5% for rifabutin-based triple therapy. They used the Pylera^®^ regimen regardless of resistance pattern and achieved eradication rates comparable to those of the tailored therapies, and its efficacy decreased only when used as a fourth line of treatment. They argued that this regimen can be used successfully as second- or third-line therapy without resorting to bacterial culture. Unfortunately, side effects occurred more frequently in patients taking Pylera than in patients receiving other therapies (they occurred in more than 30% of cases, with more frequent premature discontinuation of therapy) [21].

In a 2017 prospective study, Fiorini et al. analyzed 116 patients with persistent *H. pylori* infection after at least one eradication therapy trial who were treated with Pylera^®^ four times daily and esomeprazole 20 mg twice daily for 10 days. The Pylera^®^ regimen achieved an eradication rate of 81.0% in the ITT analysis and 87.0% in the PP analysis. The cure rate remained high until used as the fourth line of therapy, while it decreased to a low level (67%) in patients with more than four treatment failures. In this study, treatment adherence was good in more than 90% of patients. With the exception of dysgeusia and stool discoloration, a total of 71 (65.7%, 95% CI: 56.4–74.0) patients complained of at least one adverse effect, mainly gastrointestinal symptoms, fatigue, and asthenia. All symptoms were classified as mild to moderate in severity and resolved after cessation of therapy. No serious adverse events occurred. The authors concluded that bismuth-based quadruple therapy is effective as rescue therapy for patients infected with multidrug-resistant *H. pylori* strains [119].

In a 2016 prospective study, Fiorini et al. proposed therapy with esomeprazole 40 mg bid, amoxicillin 1 g bid, and rifabutin 150 mg die for 12 days to 257 treatment-experienced patients infected with a triple-resistant (clarithromycin, levofloxacin, and metronidazole) *H. pylori* strain. All patients reported good compliance with therapy. Overall, the infection was eradicated in 213 patients, with a cure rate of 82.9% in the ITT analysis and 88.7% in the PP analysis. The number of treatment discontinuations did not affect the eradication rate. A total of 44 (18.3%) patients complained of side effects, including nausea/vomiting (6 cases), abdominal pain (13 cases), mild diarrhea (12 cases), headache (4 cases), pruritus (4 cases), taste disturbance (4 cases), and myalgia (1 case). No case of leukopenia was observed in this series. Thus, this 12-day combination of low-dose rifabutin and high-dose proton pump inhibitor proved to be a safe and reliable option for treatment-experienced patients infected with triple-resistant strains [120].

Saracino et al. (2020), in a retrospective analysis of data collected between January 2016 and December 2019, compared the outcome of dyspeptic patients with at least one treatment failure who received either 12 days of rifabutin-based triple therapy (esomeprazole 40 mg and amoxicillin 1 g, both twice daily, and rifabutin 150 mg once daily) or 10 days of quadruple therapy with Pylera and esomeprazole 20 mg b.i.d. A total of 270 patients were treated with rifabutin-based therapy, and the overall eradication rate was 61.9%. Pylera^®^ therapy was administered to 153 patients, and the cure rate was 88.3%. Depending on the number of prior therapy attempts, the eradication rate for rifabutin-based therapy was 68.3% in the second-line setting and further decreased to 63.1% in the fourth-line setting. With Pylera^®^ therapy, the cure rate was 94.8% in the second-line treatment and remained at 89.6% in the fourth-line treatment, then declined again. In patients receiving rifabutin-based triple therapy, at least one adverse event was reported in 46.4% of cases (105/226). The most common adverse events were diarrhea (9.3%), abdominal pain (8.8%), nausea (7.7%), headache (6.6%), and dyspepsia (6.0%). Three patients (1.3%) discontinued therapy due to adverse events, one of whom had already taken 90% of the antibiotics and was therefore included in the follow-up. Patients treated with Pylera^®^ reported at least one adverse event in 65.5% of cases (95/145). The most common adverse events were nausea (29.7%), drowsiness (24.1%), asthenia (22.8%), dyspepsia (19.3%), and diarrhea (17.9%). In eight patients, treatment could not be continued (5.2%) due to side effects. This study demonstrates the high efficacy of Pylera^®^ as salvage therapy compared to rifabutin-based therapy [121].

In a 2018 study, Mascellino et al. analyzed data from forty patients infected with *H. pylori* who had previously been treated with one or more eradication attempts and underwent upper endoscopy at an academic hospital in Rome. They analyzed resistance to the main antibiotics used and the eradication rate of culture-specific therapy compared with empiric therapy with Pylera^®^, a rifabutin-based triple, or a levofloxacin-based triple. The eradication rate (68%) of culture-specific therapy in these patients was not different from that after empirical therapy (82%). This may be because the bacteria in the 10 empirically treated patients in whom *H. pylori* did not grow in vitro were present in a less virulent or quiescent phase or in very low numbers that could be cultured. The authors did not provide data on the eradication rate of each therapy [113].

The same group retrospectively studied a group of patients (80) with upper gastritis who had failed prior first-line treatment and had a positive UBT or endoscopy at the same hospital in Rome. They investigated secondary resistance of *H. pylori* to the most commonly used antibiotics mentioned above. The eradication rate in this population by quadruple therapy based on PPI (omeprazole 20 mg), bismuth (220 mg bid), MZ (400 mg tid), and TE (250 mg tid) was 90% (72/80). They showed that this therapy was also effective in this population [114].

Losurdo et al. analyzed 73 consecutive patients who had failed first-line therapy and were referred to their center between January 2017 and 2020 in a prospective, non-randomized, open-label study. Triple therapy with amoxicillin and clarithromycin had failed in 55 patients and sequential therapy in the remaining 18. Patients received ten days of full-dose triple therapy with BQT Pylera^®^ in combination with a PPI of the investigator’s choice. Seventy-two patients took at least 90% of the tablets; only one patient did not complete therapy due to side effects (nausea and diarrhea), resulting in eradication failure. In the ITT analysis, BQT was successful in 62 patients (eradication rate 84.9%). In the PP analysis, the eradication rate was 86.1%. Adverse events were observed in 14 subjects (20.5%), mainly diarrhea (six patients) and weakness (six patients). Nausea (two patients), dysgeusia (two patients), and headache (two patients) occurred less frequently. Adverse events did not affect eradication outcomes (*p* = 0.39). This report confirms that BQT is an effective second-line treatment [122].

In another study, Di Ciaula et al. administered Pylera^®^-based therapy (plus omeprazole 20 mg cp bid) to 142 treatment-experienced patients, with no eradication occurring in only five cases (one patient in second-line treatment and four patients in fourth-line treatment) and two patients discontinuing treatment. Overall, minor adverse events occurred in 5% of patients who received eradication therapy. They also used levofloxacin-based therapy (PPI + amoxicillin 1000 mg + levofloxacin 250 mg, each administered twice daily for 10 days) as second-line therapy and achieved an eradication rate of 57.1% in this group of treatment-experienced patients [116].

**Table 11:** Eradication Rate of bismuth-based regimens in treatment experienced patients.

Antibiotic Regimen	Year	Eradication Rate %	Region	Research Group	Ref.
Bismuth-based quadruple therapy (Pylera^®^)	2020	86.8% at ITT analysis, 90% at PP analysis,	Emila Romagna	Saracino et al.	[21]
Bismuth-based quadruple therapy	2020	90% (not indicated percentages at ITT and PP analysis)	Lazio	Mascellino et al.	[114]
Bismuth-based quadruple therapy (Pylera^®^)	2017	96.7% at ITT analysis, 97.8% at PP analysis,	Puglia	Di Ciaula et al.	[116]
Bismuth-based quadruple therapy (Pylera^®^)	2017	81% at ITT analysis, 87% at PP analysis,	Emilia- Romagna	Fiorini et al.	[118]
Bismuth-based quadruple therapy (Pylera^®^)	2020	88.3%(not indicated percentages at ITT and PP analysis)	Emilia- Romagna	Saracino et al.	[121]
Bismuth-based quadruple therapy (Pylera^®^)	2022	84.9% at ITT analysis, 86.1% at PP analysis,	Puglia	Losurdo et al.	[122]

Abbreviations: ITT, Intention to Treat; PP, Per Protocol.

**Table 12:** Eradication Rate of other regimens in treatment experienced patients.

Antibiotic Regimen	Year	Eradication Rate %	Region	Research Group	Ref.
Clarithromycin-based sequential	2020	84.2% at ITT analysis, 91.4% at PP analysis	Emilia- Romagna	Saracino et al.	[21]
Levofloxacin-based triple therapy	2020	80.4% at ITT analysis, 86.6% at PP analysis	Emilia- Romagna	Saracino et al.	[21]
Rifabutin-based triple therapy	2020	75.6% at ITT analysis, 83.8% at PP analysis	Emilia- Romagna	Saracino et al.	[21]
Levofloxacin-based triple therapy	2017	57.1% (not indicated percentages at ITT and PP analysis)	Puglia	Di Ciaula et al.	[116]
Rifabutin-based triple therapy	2016	82.9% at ITT analysis, 88.7% at PP analysis	Emilia- Romagna	Fiorini et al.	[120]
Rifabutin-based triple therapy	2020	61.9% (not indicated percentages at ITT and PP analysis)	Emilia- Romagna	Saracino et al.	[121]

Abbreviations: ITT, Intention to Treat; PP, Per Protocol.

## 10. Discussion

The World Health Organization (WHO) has warned of the importance of antimicrobial resistance, one of the major health issues of our century [123]. In 2014, the WHO published the first global report on antimicrobial resistance surveillance, providing an initial picture of the scale of the problem and showing that there is a lack of adequate surveillance in many regions of the world and major gaps in knowledge about common pathogens of major public health importance [124].

The origin of antibiotic resistance is multifactorial. Some bacteria may have an intrinsic predisposition to develop resistance in their genome that has evolved over time [125]. The spread of antibiotic resistance is a natural process that would occur even without human intervention. Since the discovery of the first antibiotic, bacteria have evolved various mechanisms to counteract the effects of antibiotics. Selection pressure from antibiotics reinforces the development of antibiotic resistance by enabling microbial adaptation mechanisms and the natural selection of more resistant bacteria [125]. The overuse and misuse of antibiotics in various fields, from human medicine to animal husbandry to agriculture, has contributed to the spread of antibiotic resistance [125,126]. In many developing countries, antibiotics are widely used without a doctor’s prescription, which is associated with inappropriate duration and choice of drug and dose. On the contrary, in developed countries, antibiotics are overprescribed because of patient expectations and uncertain diagnoses [127,128].

The Centers for Disease Control and Prevention (CDC) in the USA estimates that 30% to 50% of all antibiotics prescribed in outpatient clinics are unnecessary, and 30% of antibiotics used in hospitals are prescribed without indication and incorrectly [129]. The food and animal industries have also contributed to the overall problem of antibiotic resistance. In the past, antibiotics were used not only to treat animal diseases, but also to prevent infections and as growth promoters, both in the United States and in Europe. Since 2006 the European Union has banned antibiotic use for animal growth promotion. The USA has reduced veterinary antibiotic use without prescription in veterinary medicine, while in developing countries the use of antibiotics for these purposes is still a common practice [126].

Data collected in Italy over the past five years show high rates of primary antibiotic resistance to clarithromycin, levofloxacin, and metronidazole. This is consistent with data collected in other European and foreign countries. Megraud et al. in 2018 analyzed the antibiotic susceptibility data of 1211 *H. pylori*-positive adult patients from 18 European countries according to a standardized protocol. The resistance rate for clarithromycin, levofloxacin, and metronidazole was above the 15% threshold of resistance recommended for susceptibility testing. Resistance rates were significantly higher in Central/Western and Southern than in the Northern European countries, with Italy having the highest rates (36.9% for clarithromycin and 29.2% for levofloxacin). They found a significant association between *H. pylori* clarithromycin resistance and consumption in the community of macrolides and between levofloxacin resistance and consumption of quinolones [13]. Data on 2852 *H. Pylori*-infected treatment-naive patients from European Registry on *H. pylori* Management (Hp-EuReg) are similar [130]. The antibiotic resistance rate against clarithromycin, levofloxacin, and metronidazole is high even in the USA and in the Asia/Pacific area, with low rates of resistance against amoxicillin and tetracycline [131,132]. However, the resistance rates in Italy seem to be the highest in Europe. Megraud et al. linked clarithromycin and levofloxacin resistance to the wide consumption of these antibiotics in the community [13]. In fact, they are often prescribed to treat other common infections in the general population (e.g., respiratory, genital, and urinary infections, parasite infestation) [133]. In the period 2000–2010, global macrolide consumption increased by 19% and fluoroquinolone consumption by 64% [134]. The high primary resistance rate registered for metronidazole in developing countries could be related to its massive use to treat parasite infestations in these regions [132,133].

In Italy, the eradication rates of clarithromycin-based quadruple therapy are still enough satisfactory for the treatment of naive patients (eradication rates always >85% at both ITT and PP analysis, with not all studies reaching the threshold of 90%). A clarithromycin-based quadruple therapy could be an effective first-line treatment even if current European guidelines recommend a bismuth-based therapy in countries with high rates of both clarithromycin and metronidazole resistance [17].

Saracino et al., Fiorini et al., and Gatta et al. reported a double resistance rate for clarithromycin and metronidazole in naive patients above 15% [108,109,110].

According to these findings, clarithromycin-based quadruple therapies should not be prescribed in Italy. On the contrary, according to recent guidelines of the Italian society of gastroenterology (SIGE) and the Italian society of digestive endoscopy (SIED), there is consistent evidence that a clarithromycin-based quadruple could be considered a good option for the first-line treatment of *H. pylori* infection in Italy. The authors performed an analysis of the current evidence from Italy and found that both types of clarithromycin-based quadruple therapies seem to perform well in Italy, providing eradication rates of around 90%. Bismuth quadruple therapy should be preferred in subjects who have previously received clarithromycin for conditions other than *H. pylori* infection [135]. American College of Gastroenterology guidelines recommends either a bismuth quadruple therapy or a concomitant therapy with PPI, clarithromycin, amoxicillin, and metronidazole as first-line regimens [18]. The same recommendations were made by The Toronto Consensus for the Treatment of *Helicobacter pylori* Infection in adults [19].

The studies made in our country demonstrated a satisfactory eradication rate for bismuth-based regimens when used either for first or second line. When talking about rescue regimens different from the BQT, they do not always demonstrate a similar efficacy. In the last 5 years, in Italy, the efficacy of levofloxacin-based triple ranged from 57.1% to 84.4%. This is in line with other studies [136,137]. These data are completely different from the past; in fact, our group obtained good results (eradication rates always >85%) with the use of levofloxacin and moxifloxacin-based regimens for both treatment-naive and treatment-experienced patients [138,139,140].

This is due to the rise of quinolone resistance in the last years. However European, Canadian, American, and recent Italian guidelines still recommend it as second-line regimen after failure of BQT regimens [17,18,19].

The efficacy of rifabutin-based triple ranged from 61.95 to 88.7% in Italy. European guidelines recommend rifabutin as a second- or third-line regimen after failure of BQT. The same recommendation is made by the American guidelines while according to the Canadian ones Rifabutin regimens should be restricted to patients who have failed to respond to at least three prior options [17,18,19].

Italian guidelines recommend this regimen only after failure of multiple regimens because of the potential bone marrow toxicity, the utility for treating mycobacterial infection in patients with HIV, and a very high cost. Another salvage regimen after multiple failures could be the dual therapy with PPIs and high-dose amoxicillin for 14 days (i.e., omeprazole or esomeprazole 40 mg and amoxicillin 1 g, both three times daily), which has been shown to achieve high cure rates [135].

Italian guidelines bring another innovation. The authors recommend targeted therapy based on culture and in vitro antimicrobial susceptibility testing after failure with three lines of treatment, and no more after two. This is because the efficacy of “tailored” therapy has shown inconsistent results in the literature [135]. Saracino et al. administered the Pylera^®^ regimen regardless of resistance pattern and achieved eradication rates comparable to those of the tailored therapies, while according to Mascellino et al. the eradication rate (68%) of culture-specific therapy in experienced patients was not different from that after empirical therapy (82%). These data are in line with the findings of the authors of the guidelines mentioned above. 

*H. pylori* antibiotic resistance is a serious problem worldwide. Fallone et al. in 2019 proposed some solutions to contain the problem: the development of more easily accessible methods of resistance testing, such as biomarker analysis of stool samples, substituting vonoprazan for proton pump inhibitors, adding probiotics to reduce the side effects of antibiotics and vaccine development [103]. Our research group in the past tried to find other molecules to treat *H. pylori* infection. A trial with rifaximin plus either clarithromycin or levofloxacin and a PPI showed optimal compliance but a limited eradication rate in adult patients when compared to standard first-line treatment [140].

## 11. Methods

A review of the literature was performed with the purpose of finding papers focusing on Helicobacter Pylori antibiotic resistance in Italy through August 2022. The electronic databases searched included PubMed, Medline and Google Scholar, Scopus, and Embase. We focused on the following keywords and terms: (“Helicobacter pylori”) AND (“antibiotic resistance” OR “treatment eradication rates” OR “Italy”. We sought these terms within the title, abstract, and keywords. 

Studies included in this review were carefully reviewed by 2 authors. We included all papers with full text available. Exclusion criteria were language other than English and Italian; availability only of abstracts; reviews; metanalysis; case reports and case series. Sixteen articles met the inclusion criteria and are analyzed here. This section may be divided by subheadings. It should provide a concise and precise description of the experimental results, their interpretation, as well as the experimental conclusions that can be drawn.

## 12. Conclusions

To conclude, our review highlighted some key points: in Italy, the prevalence of both primary and secondary resistance of HP to clarithromycin, metronidazole, and levofloxacin is >15%, the common threshold for choosing alternative empiric regimens; even if rates of primary antibiotic resistance to clarithromycin and metronidazole are high (even dual resistance seems to be >15%) N-BQT seem to be effective in naive Italian patients; BQT is effective as primary and rescue therapy even if used as third-line therapy and resistance to tetracycline, amoxicillin, and rifabutin in Italy at the moment remains low, so these antibiotics still represent an alternative only as a rescue treatment in selected cases.
